# Quantitative analysis and clinical application of iris circulation in ischemic retinal disease

**DOI:** 10.1186/s12886-021-02165-1

**Published:** 2021-11-15

**Authors:** Yanwen Jia, Wenwen Xue, Xiaowei Tong, Yulan Wang, Lipu Cui, Haidong Zou

**Affiliations:** 1 Department of Ophthalmology, Shanghai General Hospital Affiliated Nanjing Medical University, 180 Kangding Road, Jing An District, Shanghai, 200000 China; 2 Shanghai Eye Diseases Prevention & Treatment Center, Shanghai Eye Hospital, Shanghai, China; 3 Department of Ophthalmology, Changzhou Second People’s Hospital Affiliated Nanjing Medical University, Changzhou, China; 4 Shanghai Key Laboratory of Fundus Diseases, Shanghai, China; 5 National Clinical Research Center for Eye Diseases, Shanghai, China; 6 Shanghai Engineering Center for Precise Diagnosis and Treatment of Eye Diseases, Shanghai, China

**Keywords:** Iris vessel, Ischemic disease, Optical coherence tomography angiography, Retinal vein occlusion, Diabetic retinopathy

## Abstract

**Background:**

To evaluate quantitative changes in iris blood circulation in patients with ischemic risk.

**Methods:**

This observational case-control study included 79 patients with diabetic retinopathy (DR) and retinal vein occlusion (RVO). The RVO group included 21 patients; the monocular proliferative diabetic retinopathy (PDR) group included 19 patients; the nondiabetic retinopathy (NDR) group included 18 patients; and the healthy control group included 21 healthy controls. In the RVO group, we analyzed RVO affected eyes, RVO contralateral eyes, and healthy control eyes. We also compared eyes with PDR and contralateral eyes without PDR, patients with diabetes mellitus (DM) without DR, and healthy control eyes. The microvascular networks of the iris and retina were analyzed using optical coherence tomography angiography. The analysis included vessel area density (VAD) and vessel skeleton density (VSD) of iris and retina.

**Results:**

In the RVO group, the VAD and VSD of iris in the affected eye were higher than those in contralateral and healthy control eyes, and the VAD and VSD of contralateral eyes were higher than those of healthy control eyes. The retinal blood flow of the RVO eyes was less than that of the contralateral and healthy control eyes, but there were no difference between the contralateral eyes and healthy control eyes. The VAD and VSD of iris in PDR were larger than nonproliferative diabetic retinopathy (NPDR) and the NPDR were larger than NDR. There were no differences between NDR and healthy control eyes. Also, there were no differences among the four groups with respect to retinal blood flow.

**Conclusions:**

Compared with the retina, iris blood circulation quantitative analysis data seem to be more sensitive to ischemia and may be used as a new predictor of ischemic disease, even if further research is needed to better understand the clinical value and importance of this analysis.

**Trial registration:**

The trial is registered with the clinical trial registration number nct03631108.

## Background

Diabetic retinopathy (DR) and retinal vein occlusion (RVO) are two of the most common retinal ischemic diseases and cause severe visual impairment in millions of people worldwide [1]. The common mechanism of these two diseases is retinal vascular lesions, resulting in ischemia, hypoxia, and the formation of retinal neovascularization in severe cases. In some patients, neovascularization can appear at the angle of the chamber and surface of the iris [2] and develop into neovascular glaucoma (NVG). NVG is a disease with a high incidence of blindness [1, 3]. At present, intravitreal anti-vascular endothelial growth factor (VEGF) therapy and retinal photocoagulation have a certain curative effect against retinal neovascularization and macular edema secondary to ischemia, but the occurrence of iris neovascularization (NVI) cannot be reduced using these two methods [4]. The regression effect and lowering intraocular pressure of the occurring NVI are also temporary [5], and NVG can only be delayed but not prevented [6]. There are some limitations in the treatment of iris vascular complications caused by ischemic diseases; thus, it is very important to detect iris vascular changes in the early stage for the treatment and prognosis of ischemic diseases.

In the past, some scholars thought that there were changes in the iris blood vessels of patients with ischemic diseases and some scholars believed that the changes in iris blood vessels occurred earlier than those in the retina. For example, Demeler et al. [7] performed iris angiography for 75 patients with diabetes mellitus (DM) and concluded that almost all patients with DR had spot dye leakage in iris vessels and speculated that the damage to the iris blood vessels in patients with DM was earlier than that to the retinal vessels. Michel et al. [8] analyzed the history of four patients with ischemic RVO and concluded that close follow-up should be conducted if iris vasodilation occurs in eyes with ischemic RVO. They believed that such patients are prone to NVI. Davide et al. [9] proposed that if iris blood vessels have changed before retinal neovascularization, the retinal disease is at an early stage of DR and RVO. In the past, these studies were based on qualitative observation of iris vessels using angiography. Angiography lacks objective and quantitative evaluation of iris vascular changes, and it is an invasive examination that is not suitable for the screening and follow-up of patients at risk of ischemic disease. Iris blood vessels can be visualized and quantitatively analyzed using optical coherence tomography angiography (OCTA) of the anterior segment. In our study, to explore the early quantitative changes and clinical value of iris blood circulation in patients with ischemic disease, the iris blood circulation indexes of patients with RVO and DR with ischemic factors, their contralateral eyes, and eyes in a healthy control group were quantitatively analyzed using OCTA.

## Methods

This was an observational case-control study. This study was approved by the Ethics Committee of the First People’s Hospital Affiliated to Shanghai Jiaotong University (ethical approval number: 2018ky181). All experiments on human subjects were conducted in accordance with the ethical standards stipulated in the Declaration of Helsinki. Informed consent was obtained from all subjects before examination. The trial is registered with the clinical trial registration number nct03631108.

### Inclusion and exclusion criteria

This study included patients who visited Shanghai Eye Disease Prevention and Control Center/Shanghai Eye Hospital between January 2020 and April 2020.

The inclusion criteria were as follows: (1) age ranging from 18 to 80 years; (2) RVO group: fundus fluorescein angiography (FFA)-based diagnosis of ischemic RVO in one eye, i.e., there were more than 10 optic disc areas without retinal perfusion [10], including central RVO and branch RVO, and no ischemic or hemorrhagic fundal lesions were found in FFA of the contralateral eye; (3) monocular PDR group: FFA-diagnosed monocular PDR and contralateral nonproliferative diabetic retinopathy (NPDR) (according to the National Academic Conference of Funds Diseases in 1985); (4) nondiabetic retinopathy (NDR) group: DM was diagnosed and matched in terms of age and sex with the monocular PDR group; FFA examination revealed no DR changes in the bilateral fundus; (5) healthy control group: patients in whom bilateral eye examination revealed no obvious abnormalities and with no history of diabetes, hypertension, or other systemic diseases, matched with the age and sex of patients with the monocular RVO and monocular PDR groups; and (6) normal intraocular pressure (10–21 mmHg).

The exclusion criteria were as follows: (1) previous history of uveitis, iris tumor, NVI, or cyanosis; (2) history of anti-VEGF or intravitreal steroid therapy; (3) history of retinal laser photocoagulation; (4) history of internal eye surgery;(5) high myopia and hyperopia; and (6) poor fixation and compliance.

All subjects underwent routine ophthalmic examinations, including uncorrected visual acuity, computer optometry (ARK-1 s, Nidek, Japan), best-corrected visual acuity, noncontact intraocular pressure (NT-510, Nidek, Japan),the conjunctiva, cornea, anterior chamber, pupil, iris, lens, vitreous and retina examination, fundus examination, digital photography (Topcon NW300, Topcon, Japan), iris and macular blood flow scanning (Cirrus HD-OCT 5000, Carl Zeiss Meditec, Dublin, CA) were analyzed. FFA fundus angiography was performed in both the monocular RVO and monocular PDR groups.

### OCTA iris blood flow evaluation

**Fig. 1:**
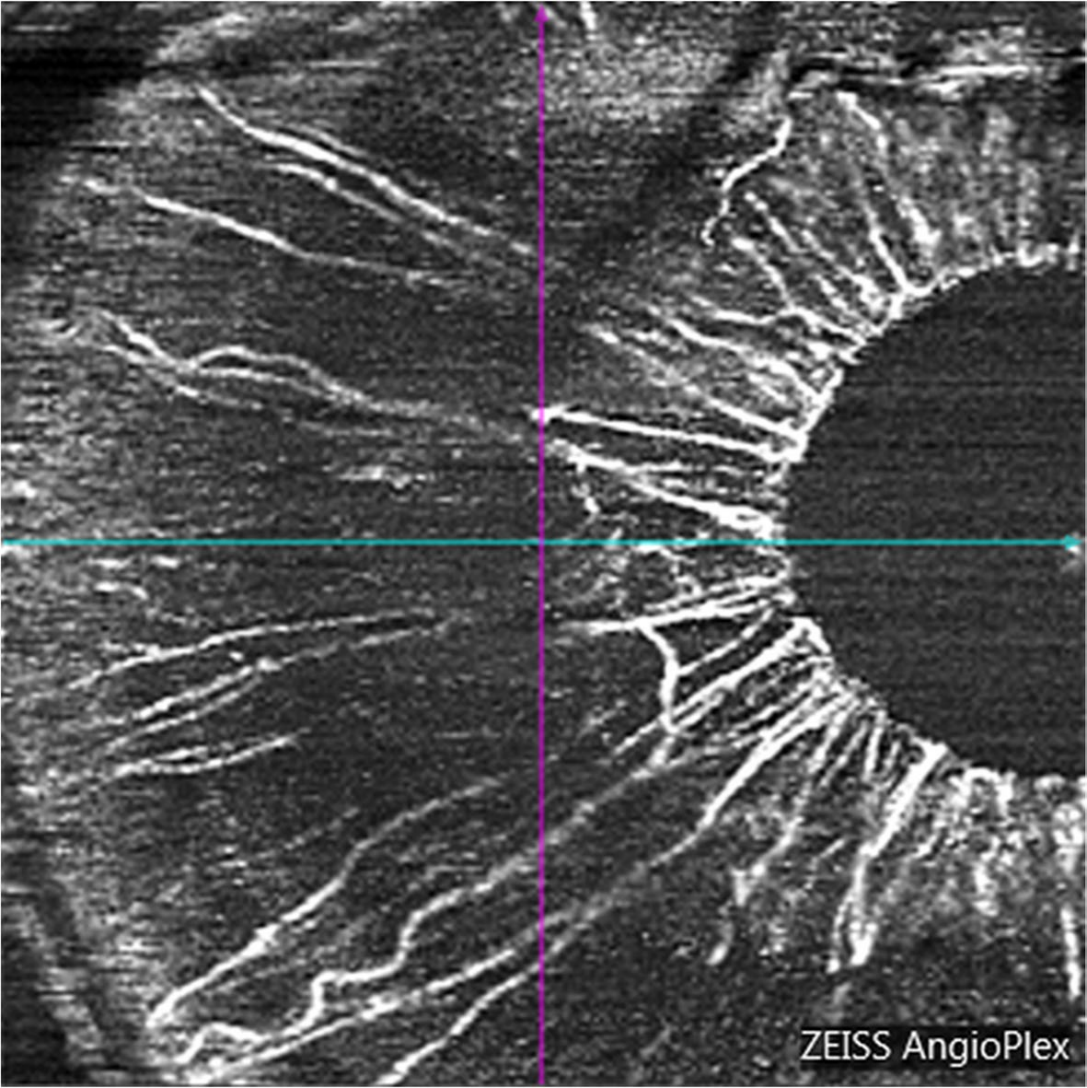
Iris OCTA for a healthy eye, male, 47 years old

**Fig. 2:**
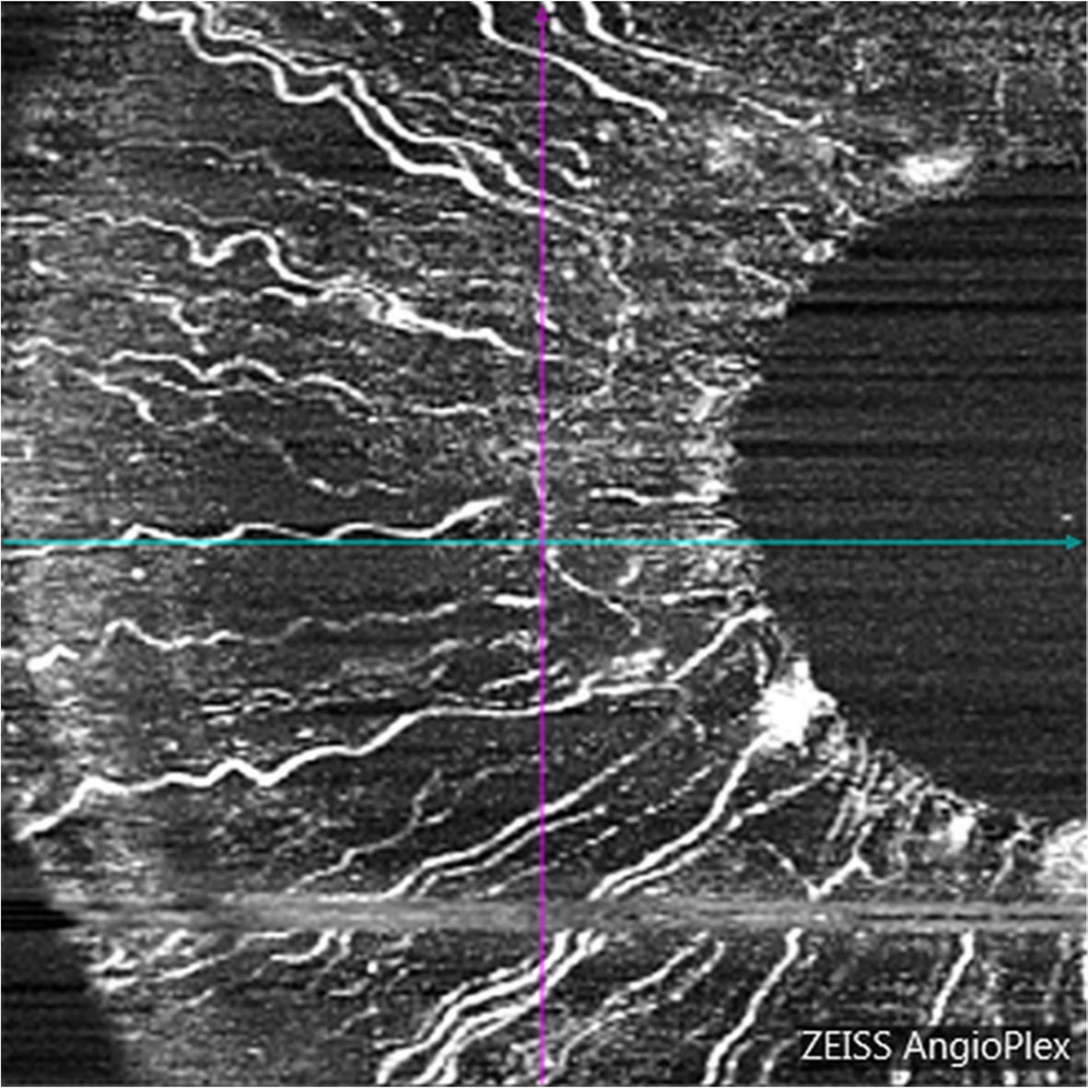
Iris OCTA for a PDR eye, male, 48 years old

Using the CIRRUS HD-OCT 5000 (Carl Zeiss, Meditec, Dublin, CA) plus a + 20D anterior segment lens, we selected the anterior segment vascular mode and scanned a dimension of 3 × 3 mm. All subjects were scanned under the same light source without pupil dilation. The focus was adjusted until the texture of the iris surface was visible, and the horizontal distance of the scan frame was from the center of the pupil to the 9 o’clock position of the corneal limbus (Fig. 1 and 2). The patients were asked to avoid frequent blinking. We calculated the vessel area density (VAD) and vessel skeleton density (VSD) of the iris using the optical microangiography method and MATLAB software (R2017a; MathWorks Inc., Natick, Massachusetts, USA). VAD is the ratio of the total image area occupied by blood vessels to the total image area, and VSD is the ratio of the length occupied by blood vessels to the total image area [11]. The region of this study were as follows: (1) vertical distance was based on the 9 o’clock position of the pupil margin above and below 150 pixel values; (2) horizontal distance was 150 pixel values from the 9 o’clock position of the pupil margin to the corneal limbus; (3) horizontal distance was divided into 3 equal parts, the 1/3 segment, 2/3 segment, and full segment. We calculated each value three times and obtained the average.

### Retinal blood flow evaluation

Retinal blood flow was examined as follows: at the end of iris scanning, the anterior segment lens was removed, the macular blood flow mode was selected, the scanning range was 6 × 6 mm, and the macular fovea was placed in the center of the scanning range. The full-thickness image of retinal blood flow was loaded into MATLAB software, and the mean blood flow density (VAD) and mean blood flow density (VSD) of retinal blood vessels were calculated using the optical microangiography method.

### Statistical analysis

For α = 0.05, power (1 − β) = 0.8, expected maximum difference between groups of 0.1, and standard deviation of 0.05, each group required at least six samples [12].

In the DM control group and healthy control group, one eye was taken (we selected the patient’s eyes according to the random principle), and the results were statistically analyzed. Statistical analysis was performed using SPSS 16.0 software. Count data are expressed as a percentage (%), and measurement data are shown as mean ± standard deviation ($\overline{x}$ ± SD). Measurement data were normally distributed as assessed using the Kolmogorov–Smirnov test, and homogeneity of variance was confirmed using the Levene test. The data were compared between two groups using an independent sample *t*-test, and data were compared among multiple groups using one-way analysis of variance. Categorical data were compared using the chi-square test. *P* < 0.05 was considered statistically significant.

## Results

### General information on patients

This study included 79 patients, including 21 patients in the monocular RVO group (12 women, mean age: 56.67 ± 10.96 years), 19 patients in the monocular PDR group (9 women, mean age: 57.05 ± 13.02 years), 18 patients in the NDR group (10 women, 54.72 ± 13.20 years) and 21 healthy controls (13 women, mean age: 54.05 ± 14.17 years. No significant differences in age or sex were observed among the RVO, PDR, NDR, and healthy control groups (*P* > 0.05).

### Comparison of VAD and VSD in different iris segments in RVO eyes

VAD and VSD in the 1/3 iris segment of eyes with RVO were greater than those in the 2/3 iris segment and full range, respectively, and the differences were statistically significant (*P* < 0.05). VAD and VSD in the 2/3 iris segment were greater than those in the full segment, but the differences were not statistically significant (*P* > 0.05).

### Comparison of VAD and VSD in different iris segments in PDR eyes

VAD and VSD in the 1/3 segment of iris in eyes with PDR were significantly greater than those in the 2/3 segment and full segment, respectively (all *P* < 0.05). VAD and VSD in the 2/3 segment were significantly greater than those in the full segment (all *P* < 0.05).

### Comparison of the degree of deviation of VAD and VSD values in different iris segments in different eyes

**Fig. 3:**
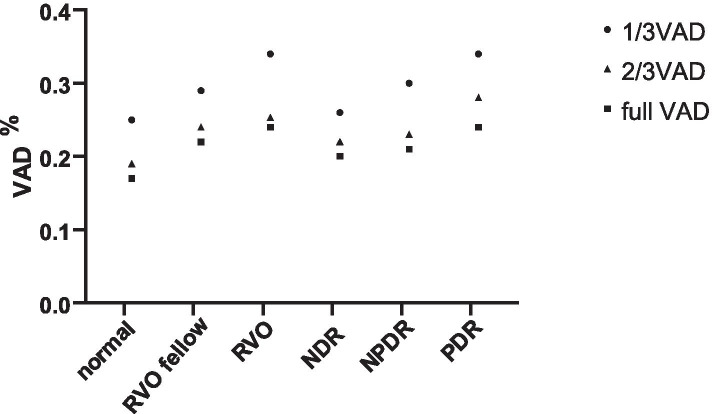
Trends of changes in iris vessel area density (VAD) in different segments in different eyes

**Fig. 4:**
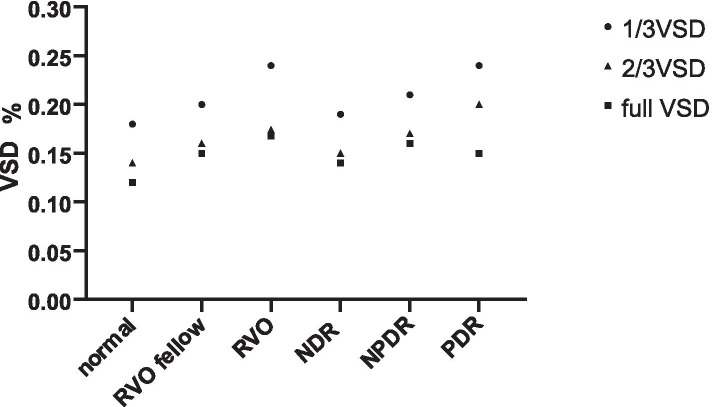
Trends of changes in iris vessel skeleton density (VSD) in different segments in different eyes

Figures 3 and 4 show the average VAD and VSD values of the iris in different segments for all subjects. The average VAD and VSD values of the 1/3 iris segment were highest, and the degree of deviation is larger than that of the 2/3 and whole iris segment. Considering the anatomic structure, the blood vessels in the 1/3 range of the iris are the densest, which can reflect the quantitative changes in iris blood circulation more intensely than the 2/3 segment and the whole segment. Therefore, we selected VAD and VSD values in the 1/3 segment of iris for analysis.

### Comparison of retinal blood flow and 1/3 iris VAD and VSD values among RVO, RVO contralateral, and healthy control eyes

Significant differences in the 1/3 iris VAD and VSD values were observed among RVO, RVO contralateral, and healthy control eyes (all *P* = 0.000). Furthermore, pairwise comparison showed that the VAD and VSD values of the RVO eyes were significantly higher than those of the contralateral and healthy control eyes (*P* < 0.05). The VAD and VSD values of contralateral eyes were higher than those of healthy eyes, but the difference was not statistically significant (*P* > 0.05). The retinal blood flow values in RVO eyes were significantly lower than those in contralateral and healthy control eyes (all *P* < 0.05), but no statistical difference was observed between the contralateral and healthy control eyes (all *P* > 0.05) (Table 1).

**Table 1:** Comparison of retinal and 1/3 iris VAD and VSD in RVO, RVO contralateral, and healthy control eyes

	RVO eye	RVO contralateral eye	Healthy control eye	F	P	P1	P2	P3
Iris 1/3 VAD	0.34 ± 0.07	0.29 ± 0.07	0.26 ± 0.03	11.191	0.000	0.008	0.000	0.052
Iris 1/3 VSD	0.24 ± 0.06	0.20 ± 0.05	0.18 ± 0.03	8.820	0.000	0.010	0.000	0.145
Retina VAD	0.51 ± 0.03	0.53 ± 0.04	0.53 ± 0.01	3.487	0.037	0.031	0.022	0.891
Retina VSD	0.13 ± 0.01	0.14 ± 0.01	0.14 ± 0.01	7.823	0.001	0.028	0.000	0.096

P, comparison of all three groups; P1, RVO eye and contralateral eye; P2, RVO eye and healthy control eye; P3, contralateral eye and healthy control eye; *P* < 0.05 was considered statistically significant. F, the relative difference among the three groups

### Comparison of retinal blood flow and 1/3 iris VAD and VSD values among PDR, NPDR, NDR, and healthy control eyes

Significant differences in 1/3 iris VAD and VSD values were observed among PDR, contralateral NPDR, NDR, and healthy control eyes (all *P* = 0.000). No significant difference in VAD or VSD was observed between the NDR and healthy control eyes (all *P* > 0.05). In addition, no significant difference in VAD or VSD values was observed among the different groups (all *P* > 0.05) (Table 2).

**Table 2:** Comparison of retinal and 1/3 iris VAD and VSD in PDR, NPDR, NDR, and healthy control eyes

	PDR	NPDR	NDR	Healthy control	F	P	P1	P2	P3
Iris 1/3 VAD	0.34 ± 0.07	0.30 ± 0.05	0.26 ± 0.03	0.26 ± 0.03	12.230	0.000	0.009	0.023	0.829
Iris1/3 VSD	0.25 ± 0.05	0.21 ± 0.03	0.19 ± 0.03	0.18 ± 0.03	13.446	0.000	0.004	0.030	0.749
Retina VAD	0.51 ± 0.04	0.52 ± 0.02	0.53 ± 0.01	0.53 ± 0.01	2.113	0.106	0.260	0.564	0.629
Retina VSD	0.13 ± 0.02	0.14 ± 0.01	0.14 ± 0.01	0.14 ± 0.01	2.584	0.060	0.331	0.654	0.218

P, comparison of all four groups; P1, PDR and NPDR; P2, NPDR and NDR; P3, NDR and healthy control; *P* < 0.05 was considered statistically significant. F, the relative difference among these four groups

### Comparison of iris VAD and VSD between PDR and RVO eyes in the 1/3 segment

There were no statistical difference between PDR and RVO in 1/3 range of iris VAD and VSD (*P* = 0.838 and *P* = 0.414, respectively).

## Discussion

We observed that the blood flow of the RVO eyes was less than that of the contralateral and healthy control eyes, which was consistent with previous results [13–15]. Although previous studies have suggested that patients with RVO may have an increased risk of retinal vein occlusion in the contralateral eye compared with normal subjects [16], this study did not find any difference in retinal blood flow between the RVO contralateral and healthy control eyes. At the same time, we found for the first time that RVO eyes had significantly higher iris vascular density and blood flow density than contralateral and healthy control eyes. Hypertension is considered the main risk factor for RVO [17]. In this study, 18 (85.71%) of patients with RVO had hypertension. Hypertension affects the blood circulation system of both eyes [18], but the lesions in both eyes are not completely consistent [19]. In our study, the eyes without ischemic retinopathy showed iris blood circulation changes (the iris VAD of the contralateral eye in patients with RVO was higher than that in healthy control eyes, *P* = 0.052).

In our study, retinal blood flow density was not different in patients with different degrees of diabetes, which was inconsistent with the findings of previous literature. Most of the studies focused on the superficial retinal blood flow in the macular area with a diameter of 3 × 3 mm and found that the retinal blood flow in this area decreased with aggravation of DR. [20, 21] This study aimed to analyze the whole retinal blood flow with 6 × 6 mm area by OCT, which more comprehensively reflected the overall changes in retinal blood flow. However, large-scale research data are affected by bleeding masking and neovascularization. For the first time, we found that in patients with DM, from NDR to NPDR and then to PDR, that is, with the increase of the severity of DR, the quantitative analysis data of blood circulation such as iris vessels and blood flow also changed significantly. Theoretically, these changes indicate the existence and severity of intraocular ischemia.

Furthermore, we also found that the degree of change in iris blood circulation of the two ischemic diseases (RVO and PDR) was similar to that in normal people, and such changes were consistent with a previous study on iris fluorescein angiography [22]. The possible mechanisms were as follows: (1) Ischemic retinal releases vasoactive molecules, such as VEGF, through the vitreous to diffuse into the anterior chamber, inducing iris vasodilation [8], making the deep iris vessels covered by iris pigment dilate. The number of vessels on the surface and deep layers of the iris is increased by OCTA, that is, the VSD value is increased. The iris ciliary body itself can also produce VEGF, which can induce vascular dilation and increase iris perfusion [4]; therefore, the iris VAD value also increases. (2) Previous studies have reported that a high concentration of vasoconstrictor prostaglandin F(2) (PGF2) alpha can cause constriction of the ciliary artery in monkeys in vitro [23], whereas the concentration of PGF2 alpha in the vitreous cavity of PDR eyes is significantly reduced [24]. Therefore, we speculate that with the lower levels of PGF2 alpha in PDR eyes, the ciliary artery may expand, leading to an increase in iris VSD.

Our results suggest that in RVO and DR, the change in quantitative data of iris blood circulation seems to occur earlier than that in retinal blood circulation. In other diseases, these features of iris vessels have been confirmed. Francesco et al. [25] found that in patients with systemic lupus erythematosus, the iris blood flow of patients was higher than that in healthy controls when there were no clinical changes in the fundus. In addition, Kivlin [26] thought that iris vasodilation was a sign of retinopathy of prematurity (common ischemic retinopathy in childhood). We believe that iris vessels are more sensitive to intraocular ischemia than retinal vessels, which may be related to two different tissue structures. First, the retina is supplied by two sets of vascular systems, namely, the central retinal vessels and ciliary vessels. When our eye is ischemic, the ciliary vessel increases the blood flow to provide blood to retina without changing of retinal vessels. However, the iris is supplied only by the long posterior ciliary artery. When the eye is ischemic, the iris vessels will expand to increase the blood flow. Second, the blood flow in the retina is mainly distributed in the inner layer of the retina. The inner layer of the retina is closely connected to the outer layer, but the iris stroma is a sponge like characteristic [27]. Therefore, we speculate that when central vein occlusion or hyperglycemia leads to insufficient intraocular microcirculation perfusion, iris vessels are more likely to dilate than retinal vessel and that the iris vessels were the first to change in OCTA.

There are some limitations to our study: (1) This was an observational case-control study; for DR or RVO with obvious iris blood circulation changes, the longitudinal changes during the progression of NVI still need to be further studied. (2) Due to poor vision in patients with ischemic RVO and PDR, some patients have poor fixation. Therefore, in this study, we only included patients from whom iris quantitative analysis data could be obtained. (3) In this study, blood lipid and carotid ultrasound were not examined. These indexes may affect ocular blood. Therefore, we should include these indicators in future research. (4) Although we selected the Han population in our study, iris color is still taken into account because blood flow and the number of blood vessels detectable by iris OCTA are also affected by pigment masking [28]. Despite this study with a small sample size, we found that the application of OCTA, a noninvasive and rapid examination method, can quantitatively analyze iris blood circulation in patients with ischemic diseases, which is more sensitive to fundus ischemia than the retina. Therefore, regular observation and close follow-up of iris vessels in patients with high-risk ischemic factors and possible NVI should be performed. Through the quantitative analysis of iris blood circulation, early detection of changes in iris blood circulation, and early intervention for fundus lesions caused by ischemia and further NVI are very important for the prognosis of the disease.

## Conclusions

Compared with the retina, iris blood circulation quantitative analysis data seem to be more sensitive to ischemia and may be used as a new predictor of ischemic disease, even if further research is needed to better understand the clinical value and importance of this analysis.

## Data Availability

The data can be found in the ophthalmology department of Shanghai Eye Disease Prevention and Control Center (Shanghai Eye Hospital) Funding Sources. Further enquiries can be directed to the corresponding author.

## Abbreviations

DR: Diabetic retinopathy
RVO: Retinal vein occlusion (RVO)
PDR: Proliferative diabetic retinopathy (PDR)
DM: Diabetes mellitus
OCTA: Optical coherence tomography angiography
VAD: Vessel area density
VSD: Vessel skeleton density
NVG: Neovascular glaucoma
VEGF: Vascular endothelial growth factor
NVI: Iris neovascularization
OMAG: Optical microangiography
